# 

**Published:** 1897-08-14

**Authors:** 


### EARLY EDITION.

**Notices of Births, Deaths, and Marriages** are inserted in this column at a charge of 2s. 6d. for an announcement not exceeding four lines.

### NOTICE TO CORRESPONDENTS.

All MS., Letters, Books for Review, and other matters intended for the Editor should be addressed The Editor, "The Hospital," The Hospital Building, 28 & 29, Southampton Street, Strand, W.C.

The Editor cannot undertake to return rejected MS., even when accompanied by stamped directed envelope.

**Notice to Advertisers and Subscribers.**

All Advertisements, Orders for Copies of The Hospital, and Business Communications should be addressed to **THE MANAGER, (Not to The Editor),** The Hospital, The Hospital Building, 28 & 29, Southampton Street, Strand, London, W.C.

The Cost of Subscription, post free, is as follows:—Per week, 2½d.; per quarter, 2s. 8d.; per half-year, 5s. 3d.; per annum, 10s. 6d. Foreign:—Per annum, 15s. 2d.; payable, in all cases, in advance.

### Scraps and Gleanings.

The Hospital Saturday collection at Bedford this year has reached nearly £475.

The Bristol Hospital for Sick Children and Women during the last few years has accumulated a deficit of £668.

The Jubilee subscription to the Coventry and Warwickshire Hospital realised in donations £3,897, and in new annual subscriptions £75.

On July 8th, the Earl and Countess of Jersey distributed prizes to the sailor boys of the "Arethusa" and "Chichester" training ships.

One hundred and eighty-eight in and 835 out-patients were treated at the Weymouth and Dorset County Royal Eye Infirmary during the year ended July, 1897.

The Woking, Horsell, and Woodham Cottage Hospital received last year, £651 on the general account, and £40 for the building fund. The expenditure was £664.

In consequence of deaths and removals, the annual subscription list of the West Cornwall Infirmary, at Penzance, has already fallen off this year by more than £180.

The total cost of maintenance at the West Cornwall Miners Hospital, Redruth, in 1896 was £862, to which Lord Robartes contributed £223. 198 patients were treated.

The Goldsmiths' Company and the treasurer of the hospital, Mr. Hope Morley, have respectively sent £100 and 50 guineas to the Royal Chest Hospital, City Road, E.C.

The cost of erecting a mortuary ward in Lady Elgin's Hospital, at Gaya (Rs. 10,000), has recently been given by Raja Rameshwar Parsad Narain Singh of Mugsoodpore.

The expenditure at the Gorleston Cottage Hospital in 1896 (£179) is below the average of the past nine years. Three hundred and forty-six out and 13 in-patients were treated.

During 1896 10·38 per cent., or one out of every 9·63 of the population were treated at the dispensaries in the province of Assam, against 9·95 per cent., or one out of every 10·05 treated in 1895.

The union of the St. Mark's Ophthalmic Hospital and the National Eye and Ear Infirmary, at Dublin, has now been accomplished, the necessary Act of Parliament having received Royal assent.

It has been arranged that home visits shall be made rom the Royal Surrey County Hospital. to the indigent and infirm residing in Guildford. Two hundred home visit letters are to be issued each year.

Exclusive of £12 12s. collected at the new asylum works, Cheddleton only £50 was contributed by workpeople in the district through their workshop collection to the Leek Memorial Cottage Hospital.

Without making any deduction for the 1,056 out-patients treated during 1896, the average cost of the 566 in-patients at Hartlepools Hospital was 4s. 4d. a day. The average length of time each in-patient resided in the hospital was 17 days.

At the present time there are 100 inmates in the home connected with the Royal Alfred Aged Merchant Seamen's Institution, and 236 out-pensioners, who receive £12 a year. In 1896 £2,513 was expended on the home, and £2,962 on out pensions and special gifts.

The average cost of each patient per day at the Cleveland Cottage Hospital, Brotton, Yorkshire, during the 15 months ended December 31st, 1896, was 4s. 5·3d., against 5s. 2·7d. in 1895. Ninety-nine in-patients and 65 out-patients were treated during the same period.

During the past five years the cost per in-patient at the Cheltenham General Infirmary has been a fairly constant amount, ranging from £3 11s. 6d. to £3 19s. 1½d. Out-patients cost 3s. 8½d. in 1892, and have steadily decreased in cost until they reached 3s. 3d. in 1896.

The total payments, exclusive of the outlay on the new wing, at the Birmingham and Midland Eye Hospital last year, amounted to £5,763, against £6,133 in the previous year. A daily average of 72 in-patients were resident during the year, eac i patient being resident 17·22 days.

The Hospital Saturday collections in Hull have steadily decreased year by year since 1891, the figures being: 1891, £582; 1892, £535; 1893, £428; 1894, £531; 1895, £496; 1896, £440; 1897, £374. Street collections form the chief source of revenue of the Hull Hospital Saturday Fund!

During the past year 5,656 cases have been treated at the Manchester Southern Hospital, made up as follows: Women and Children's Hospital—Out-patients, 3,814 (994 women and 2,820 children); in-patients, 429 (146 women and 283 children). Maternity Hospital—In-patients, 180; home cases, 1,233.

Since 1893 the work done at the Scarborough Hospital has steadily increased, as the following figures show: In-patients treated in 1893, 180; in 1894, 303; 1895, 317; 1896, 354. Out-patients: 1893, 1,062; 1894, 1,212; 1895, 1,837; 1896, 2,656. Home patients: 1893, 955; 1894, 1,212; 1895, 1,406; 1896, 1,312.

We learn that Messrs. W. and A. Walker have made such a success with their antiseptic cleanser Carbolacene, to whose merits we had occasion to call attention some little time ago, that they have found it necessary to move from their old address to more commodious premises in African House, Wat r Street, Liverpool, where they have a splendid suite of new offices. Carbolacene, though a comparatively new product, has already won its way into the Government Offices and State prisons, and it will probably soon find a place in hospitals and kindred institutions.

As an illustration of the fact that the granting of free medical relief has been abused during the last few years, and has had a serious pauperising effect on the class of people who seek to obtain medical relief by ticket, the last annual report of the Sunderland Provident Dispensary gives the following figures: From 1880 to 1886 inclusive 2,932 people entered by subscribers' tickets, and from 1890 to 1896 inclusive (or 10 years later) 5,049 people entered by subscribers' tickets and infirmary orders. In the years 1880 to 1886 inclusive the number of those using tickets who became paying members equalled 34½ per cent. during the whole period. From 1890 to 1896 inclusive the percentage of ticket and infirmary order cases becoming provident members of the institution only reached 12 per cent. for the whole period, whilst last year was the worst of all, only being equal to 6½ per cent.

### The Student of Medicine.

### ROYAL COLLEGE OF PHYSICIANS AND SURGEONS, EDINBURGH.

The following questions were set in the final examination in July for the triple qualification:—

Medicine (Three questions only to be answered).

1. Describe the onset. symptoms, and course of an attack of whooping-cough. What are the chief complications which may occur?
2. What are the symptoms of angina pectoris?
3. Describe the structural changes and the clinical features of chronic hydrocephalus in a child.
4. Describe the symptoms of a typical attack of hepatic colic, due to the passage of a gall stone.

Therapeutics (All the questions to be answered, including the prescription).

1. State how hepatic colic, the result of the presence of gall stone, should be treated (1) during the attacks, and (2) in the intervals.
2. Describe the general management and the medical treatment of rheumatic fever.
3. What diet is appropriate for a severe case of diabetes?

Prescription (To be written in unabbreviated Latin, with the directions for the patient in English).

Prescribe a pill, for chronic constipation, containing aloes, nux vomica, ipecacuanha, and capsicum.

Surgery (Three questions to be answered).

1. Mention the varieties of loose bodies to be found in joints. In which joints do they usually occur, what symptoms do they produce, and how should they be treated?
2. State what you know as to flat foot, its causes, symptoms, and treatment.
3. What is fissure of the anus, its mode of formation, its symptoms, and how would you treat it?
4. What are the usual causes of enlarged glands in the neck? In what situations are such glands usually found, and how should they be treated?

Surgical Anatomy (Both questions to be answered).

1. Mention the various structures which form the spermatic cord. Where do its arteries arise, and where do its veins terminate.
2. Describe the lachrymal apparatus.

Midwifery and Gynecology (Three questions to be answered, of which the last must be one).

1. Give the clinical history of a case of tubal gestation, and indicate the circumstances under which abdominal section is called for.
2. Describe the mechanism of labour in occipito-posterior cases, and the operative interference that may be required.
3. What are the causes of asphyxia in the child at birth, and what treatment would you adopt?
4. (Gynecology.)—What are the causes, symptoms, and physical signs of retroversion of the uterus? Describe briefly the treatment of an uncomplicated case.

### BOOKS RECEIVED.

"The Lancet" Office.

"District Nursing on a Provident Basis." By Jamieson B. Hurry, M.D.

Sampson Low, Marston, and Co.

"Homburg and its Waters." By N. E. Yorke-Davies.

Hugh McDonald, Oban.

"Oban, a Health and Holiday Resort." By Edwin Baily, M.B.

**Periodicals Received.**—Medical Record, Literary Digest, London, British Realm, Industries and Iron, Medical Pioneer, Vegetarian, Edinburgh Medical Missionary Society, Guy's Hospital Gazette, Woman's Signal, Local Government Journal, Electrical Review, The New Age, The Guyoscope, Christian World, Cassell's Family Magazine, Cassell's Saturday Journal, The World of Adventure, Girl's Own Paper, Boy's Own Paper, Boy's Sunday Monthly, Sunday at Home, Zenana, Sunday Hours.

